# New insights on the association between the prostate cancer and the small DNA tumour virus, BK polyomavirus

**DOI:** 10.1186/s12967-015-0754-z

**Published:** 2015-12-23

**Authors:** Mauro Tognon, Maurizio Provenzano

**Affiliations:** Section of Pathology, Oncology and Experimental Biology, Laboratories of Cell Biology and Molecular Genetics, Department of Morphology, Surgery and Experimental Medicine, School of Medicine, University of Ferrara, 64/b, Fossato di Mortara Street, 44121 Ferrara, Italy; Oncology Research Unit, Department of Urology and Division of Surgical Research, University and University Hospital of Zurich, 8952 Zurich, Switzerland

## Abstract

In recent years the scientific literature in the field of the prostate carcinoma (PCa) pointed out on the genetic heterogeneity and mutations occurring in this tumour, while little attention was given to the causes of PCa onset, in particular infectiuos agents. In this brief commentary, we wish to point out recent advancements done on the role of the human polyomavirus BK (BKPyV) in the development of PCa by harnessing both humoral and cellular immune responses. Altogether, these new insights suggest that BKPyV is involved in the transforming activity during the multistep process of PCa development. Although these findings do not provide evidence for a causal relationship between BKPyV and PCa development, additional investigations with novel techniques will help to make it a concrete event.

In recent years the scientific literature in the field of the prostate carcinoma (PCa) pointed out on the genetic heterogeneity and mutations occurring in this tumour [1, 2], while little attention was given to the causes of PCa onset. Together with chemical and physical agents, biological agents such as viruses with oncogenic potential, which interfere with the cell cycle, are responsible for gene alterations and might be included in the putative genomic evolution of PCa.

Herein, we wish to draw the attention of the readers to the role of the human polyomavirus BK (BKPyV) in the development of PCa. BKPyV encodes two viral oncogenes, the large T antigen (Tag) and small t antigen (tag). Transformation of animal and human cells by BKPyV is operated by these two viral oncoproteins [3]. Tag binds and abolishes the functions of the tumour suppressor p53 and pRB family proteins, whereas tag interacts with the phosphatase PP2A, which activates the Wnt pathway [4]. Moreover, tag activates the phosphatidylinositol 3-kinase, an enzyme involved in pathways crucial for cell proliferation and transformation. Tag is also clastogenic and mutagenic [3]. These activities are able to hit the cellular genome, which accumulates many gene mutations/chromosome aberrations. Then, in the absence of p53 functions, due to Tag binding, the cellular DNA remains unrepaired with the consequence that the genome “derailed”. In addition, loss-of-function mutations in p53 gene at very early stages of PCa are rare [1]. Therefore, the sequestration of wild-type p53 exerted by BKPyV Tag oncoprotein at initial phases is a hallmark for BKPyV involvement [5]. These mechanisms may ensure the genetic heterogeneity of PCa.

In this context, it is worth noting that the WHO classified BKPyV as “possibly carcinogenic to human” (http://monographs.iarc.fr/ENG/Monographs/vol104/mono104.pdf) because of the sufficient evidence of its carcinogenicity in vitro and/or in vivo in animal [6]. However, at present there are not sufficient human epidemiological data to support so.

Considering that BKPyV infection is ubiquitous in the general population, it is difficult to assess a specific role of this virus in cellular transformation. BKPyV is kidney-tropic and remains latent in many human organs/tissues, including the prostate. The lifelong period of BKPyV infection, together with the fact that about 95 % of PCa are slow-growing organ-confined indolent tumors, could be responsible of those gene/chromosomal damages, which initiate the development of this malignancy. BKPyV infection has repeatedly been associated with PCa and BKPyV Tag was identified as a potential co-factor at the earliest stages of this disease [5]. Footprints of this small DNA tumour virus has been revealed in precursor inflammatory lesions [7], which may evolve in prostatic intraepithelial neoplasia (PIN) and then in overt PCa [8] Indeed, BKPyV was detected at higher prevalence in early stages PCa than in healthy control tissues, thus providing an indication for an increased risk of PCa development with the presence of BKPyV infection [9]. Notably, a peculiar cellular immune response elicited by BKPyV LTag has been observed in PCa patients with evidence of biochemical recurrence that bear BKPyV LTag positive tumors [10]. In addition, immunological data indicate that antibody response to BKPyV Tag in PCa patients at first diagnosis might associate with the clinical course of this disease [11].

Altogether, these data suggest that BKPyV Tag, as a viral oncogene, is involved in the transforming activity during the multistep process of PCa development [12]. However, additional investigations with novel techniques are needed in order to be more persuasive on the role of BKPyV in PCa.
